# Strides in Dental Education

**Published:** 1906-11

**Authors:** E. N. Kibler

**Affiliations:** Prosperity, S. C.


### STRIDES IN DENTAL EDUCATION.

By E. N. Kibler, D. D. S. Prosperity, S. C.

South Carolina Dental Association, 1906.

To gain knowledge we must study the past and the present, by so doing we can aptly foretell the future. The men who make a success and are an honor to the profession, are those who have made diligent research and are constantly striving to place the practice of Dental Surgery to the fore, men who never tire in their efforts for improvement: men whose watchword is progress.

I may say we have many men who call themselves dentists, who are mere mechanical fumblers, and who are in the profession only for the monetary consideration. Such are a disgrace to the profession and a fraud on humanity. There is no profession that has made such wonderful strides of progress as ours in the last twenty five years. We are now just blooming into the zenith of perfection.

We are standing side by side with our sister profession, medicine, and are reaping the honors that so justly belong to us, and are destined to be second to none. I believe that God in the creation of man placed within his mouth the thirty-two precious enameled jewels, as a gift for life. Have we any other organs composed of like structure. The tooth to an uneducated eye is a very simple object. But to the educated a momentous compact; what is more beautiful than the pearly luster of enamel, the protector of its sister dentine. It is needless for me to describe the three different structures and the pulp with the nerve, arteries, veins and lympathics to the educated dentist, he is fully acquainted with them.

Think of all the different compartments of vitality of the human body developed with the care, as that of a tooth and tell me it is only for a temporary period. I would sooner tell you a stone fort was built for a temporary purpose. Our mouth is the gateway of health, and our teeth the batteries that accomplish the first work of perfect digestion, that goes towards giving us a healthy body and apt mind.

To take care of these precious organs we need an educated dental surgeon. It is a high and honorable calling, that no man with high attainments and aspirings need be ashamed of. I am glad to see the diversity of progress we are making. Take the progress of Orthodontia and look at the beautifications and marks of intelligence we have given our patients.

We are now living in an age of Oslerism, when a man of broken down teeth, mal-occlusion, protrusion, etc., need not apply for a position of trust or honor, because these deformities are marks of age and idiocy to the business public. But let this man go to an ethical orthodentist who will regulate the deformities and replace the lost structures and he will then appear as a man of youth and intelligence.

I say we are meeting with honor and praise on all sides. The public is beginning to awake from a long sleep, to the object and physiological necessity of the teeth, their care and attention and it behooves us as dentists to be well informed on all subjects pertaining to our profession. The day is fast approaching when only the educated, ethical dentist will succeed; the quack will be unable to live upon the public, as that public is becoming more and more educated and will recognize him at a glance.

We cannot be too loyal to our ASSOCIATIONS. We should be proud of them and honor them by our presence. They have been the nucleus of education, from which has radiated honor, distinction and benificence. It is through our Associations we have efficient and ethical members of our State Boards, and the state Boards have stimulated the dental colleges to a higher ideal, and have run the purchasing diploma concerns out of business. We then should look forward to our annual meeting with pleasure, as a great Summer Dental School, where we exchange methods and ideas freely.

We as dentists should be a benefactor to the locality in which we reside. We should be careful in the advise we give young men who desire to study the profession.

So many seek the profession because they think it an easy road to wealth. We need no such, because usually they are the embodiment of quackery. But I thing it our duty to the profession and the public to advice young men with high moral character education and mechanical ingenuity that would be an honor to the profession and a benefaction to the public. From such we can expect the full bloom of progressiveness of the dental profession, whereby it will attain the highest recognition among the learned professions it justly deserves.

